# A conceptual and computational framework for modelling and understanding the non-equilibrium gene regulatory networks of mouse embryonic stem cells

**DOI:** 10.1371/journal.pcbi.1005713

**Published:** 2017-09-01

**Authors:** Richard B. Greaves, Sabine Dietmann, Austin Smith, Susan Stepney, Julianne D. Halley

**Affiliations:** 1 York Centre for Complex Systems Analysis, University of York, York, United Kingdom; 2 Wellcome Trust-Medical Research Council Cambridge Stem Cell Institute, University of Cambridge, Cambridge, United Kingdom; Dassault Systemes BIOVIA, UNITED STATES

## Abstract

The capacity of pluripotent embryonic stem cells to differentiate into any cell type in the body makes them invaluable in the field of regenerative medicine. However, because of the complexity of both the core pluripotency network and the process of cell fate computation it is not yet possible to control the fate of stem cells. We present a theoretical model of stem cell fate computation that is based on Halley and Winkler’s Branching Process Theory (BPT) and on Greaves *et al*.’s agent-based computer simulation derived from that theoretical model. BPT abstracts the complex production and action of a Transcription Factor (TF) into a single critical branching process that may dissipate, maintain, or become supercritical. Here we take the single TF model and extend it to multiple interacting TFs, and build an agent-based simulation of multiple TFs to investigate the dynamics of such coupled systems. We have developed the simulation and the theoretical model together, in an iterative manner, with the aim of obtaining a deeper understanding of stem cell fate computation, in order to influence experimental efforts, which may in turn influence the outcome of cellular differentiation. The model used is an example of self-organization and could be more widely applicable to the modelling of other complex systems. The simulation based on this model, though currently limited in scope in terms of the biology it represents, supports the utility of the Halley and Winkler branching process model in describing the behaviour of stem cell gene regulatory networks. Our simulation demonstrates three key features: (i) the existence of a critical value of the branching process parameter, dependent on the details of the cistrome in question; (ii) the ability of an active cistrome to “ignite” an otherwise fully dissipated cistrome, and drive it to criticality; (iii) how coupling cistromes together can reduce their critical branching parameter values needed to drive them to criticality.

## Author summary

Pluripotent stem cells possess the capacity both to renew themselves indefinitely and to differentiate to any cell type in the body. Thus the ability to direct stem cell differentiation would have immense potential in regenerative medicine. There is a massive amount of biological data relevant to stem cells; here we exploit data relating to stem cell differentiation to help understand cell behaviour and complexity. These cells contain a dynamic, non-equilibrium network of genes regulated in part by transcription factors expressed by the network itself. Here we take an existing theoretical framework, Transcription Factor Branching Processes, which explains how these genetic networks can have critical behaviour, and can tip between low and full expression. We use this theory as the basis for the design and implementation of a computational simulation platform, which we then use to run a variety of simulation experiments, to gain a better understanding how these various transcription factors can combine, interact, and influence each other. The simulation parameters are derived from experimental data relating to the core factors in pluripotent stem cell differentiation. The simulation results determine the critical values of branching process parameters, and how these are modulated by the various interacting transcription factors.

## Introduction

Stem cells present an important instance of the complexity of cellular function and gene regulation. Pluripotent stem cells possess the capacity both to renew themselves indefinitely and to differentiate to any cell type in the body. This second capacity means that the ability to direct stem cell differentiation would have immense potential in regenerative medicine. High throughput datasets are available to facilitate understanding of stem cells, but such data provide only snapshots of biological complexity, with no dynamics. Here we consider these data within a particular theoretical framework to explore their abstract dynamics, with the longer term aim to help understand cell behaviour and complexity.

Here, we suggest that in order to understand how stem-cell gene regulatory networks drive cell fate, we need to appreciate the non-equilibrium nature of regulatory networks in living cells. How can a regulatory network self-organise from one stable state of pluripotency, to another state of differentiation? Ultimately, such better understanding could aid attempts to direct such state changes. First, we need an understanding of the dynamics of such networks.

We expand on our previous theoretical framework in which self-organization and natural selection are intimate partners in non-equilibrium dynamic systems generally [1–5], and investigate an abstract model of four transcription factors (TFs) central to the self-organization of the core pluripotent network of mouse embryonic stem cells (ESCs) [6, 7]. The underlying theory of the model presented here is the Transcription Factor Branching Process (TFBP) concept explained in Halley et al. [3].

### Background

#### Complexity and modelling

The principal challenge facing the systems biology of stem cell decision-making is complexity [8]. The icon of molecular biology for the last third of the 20th century was the molecular structure of the double helix, and the central dogma of unidirectional information flow. The icon for the systems biology era may be the ‘hairball’ graph, where everything appears connected to everything else [8–10], with complex information flows and feedbacks. In the context of gene regulatory circuits, this is partly due to the often immense number of potential binding sites for key TFs in DNA. c-Myc, for example, can bind to around 30,000 sites and is a key TF in ESC circuitry, the reprogramming of pluripotency and some cancers. TF-DNA binding site data can provide information for static wiring diagram representations of regulatory circuits, but the dynamics of information flow within such circuitry remains challenging to understand.

High-throughput chromatin immunoprecipitation (ChIP)-chip [11], ChIP-seq [12] and ChIP-PET [6] experiments identify TF interactions with DNA and thereby connect TFs to putative sets of genes that they can regulate [13]. These techniques can also be used to identify a broad range of epigenetic chromatin modifications, such as methylation/acetylation status of histone proteins [14, 15] that alter the probability of TF-DNA interactions.

Large-scale data sets include complete genome sequences, dynamic measurement of gene expression levels at genome-wide scales, extensive lists of regulatory proteins and RNAs, and *in vivo* occupancy of the DNA by TFs, cofactors and nucleosomes [10]. A complete multi-layered model of embryonic stem cell circuitry, for example, will exploit such data to bridge gaps between the phenotypic behaviour of whole cells and key regulatory molecules [16].

The purpose of modelling is to abstract out some of the details of the system under study to identify likely principles of operation [17]. Computational or executable models are particularly well suited to capturing such complexity. Whereas traditional mathematical models are typically used to understand how variables relate to each other, computational models highlight interactions among system components as dynamic recipes or algorithms unfolding [17–29].

We take a complex systems approach, and use a computational model grounded in a specific theoretical framework to study abstract system dynamics. The simulation presented here, an extension of the single cistrome model of [30], exploits multiple cistrome data derived from ChIP-Seq, to ground our abstract model with physiological data sizes.

#### Problems facing attempts to model embryonic stem cell gene regulatory networks

Most attempts to model stem cell regulatory networks consider single layers of complexity, such as single TF networks, though it is important to recognise that regulatory networks span multiple organizational layers and involve many different types of regulatory elements. The ability to experimentally induce an artificial pluripotent state in differentiated cells using TFs (as demonstrated first by [31, 32]) indicates that TFs possess substantial ability to control regulatory network dynamics; Oct4 in particular stands out as a master regulator of network behaviour for several reasons (reviewed in [33]).

In contrast to the predictable patterns of development within normal embryos, some differentiation of ESCs *in vitro* can appear asynchronous and disorganized [34]. Furthermore, although we have gained substantial knowledge of component parts and their interactions within stably self-renewing ESCs, our knowledge of pluripotency exit and the control of differentiation trajectories remains fragmented. One reason for this could be that the process of pluripotency exit is itself less organized than the process of self-renewal. In other words, regulatory circuitries within individual ESCs undergoing fate computation could be fundamentally disorganized or chaotic in order to compute cell fate trajectories, a possibility explicitly captured by a recent theory of stem cell decision-making centred on ‘critical-like dynamics’ at the edge of chaos [3].

#### Review of existing attempts to model embryonic stem cell gene regulatory networks

Xu, Schaniel *et al*. note the huge amount of high-throughput data available for human ESCs [16], as well as the databases and computational tools that facilitate the study of this data. They also propose that this paves the way to *in silico* reconstruction of the regulatory networks encompassing multiple molecular layers. There are two main ways of modelling stem cell behaviour: mathematical and computational models.

Mathematical models of stem cell pluripotency consisting mostly of sets of differential equations are widely used and are reviewed more comprehensively in Eby and Colman [35]. Chickarmane et al. [36] use data from ChIP-Seq experiments on human ESCs to elucidate the architecture of transcription regulation crucial for determining cell fate–Oct4, Sox2 and Nanog are found to regulate each other as well as numerous down-stream targets. They use kinetic modelling (essentially systems of differential equations that describe the transcription of each TF gene). The authors identify what they refer to as a bi-stable switch, which arises due to the numerous positive feedback loops in the pluripotency circuitry. The switch can change state in accordance with environmental signals: if the core pluripotency TF are expressed then the switch is ‘on’, if differentiation genes are expressed, then it is ‘off’. In contrast, Herberg et al. [37] model a wider circuitry: the core pluripotency circuitry of Oct4, Sox2 and Nanog as well as FGF4/Erk and Rex1. They employ their model to investigate how proposed mechanisms and feedback regulation can account for different expression patterns in murine ESC cultures. They demonstrate that FGF4/Erk mediated negative feedback can induce molecular heterogeneity with respect to Nanog and so regulate the propensity for differentiation or loss of pluripotency.

Dunn et al. [38] create a more strictly computational model which reduces complexity and derives a set of functionally validated components and combinations of interactions that are sufficient to capture observed ESC behaviours.

Our TFBP model and associated simulation provides (i) a simplification of many of these processes in terms of a branching process model; (ii) an integration of the specific ChIP-seq mouse data, in terms of how cistromes are interconnected.

### The Transcription Factor Branching Process (TFBP)

#### Exploratory hypothesis of stem cell fate computation

The Exploratory hypothesis [3] proposes that cell fate decisions emerge when gene regulatory networks self-organize through amplification of fluctuations in the form of bursts of gene expression. Non-specific amplification of gene expression is predicted to initiate a self-organizing circuitry, where cascades of gene expression propagate and interact. However, the gene regulatory circuitry of an embryonic stem cell comprises a multitude of genetic programs that can ultimately generate multiple alternative cell phenotypes. Significantly, each lineage-affiliated genetic program should generally promote itself and repress alternatives to ensure coherence and mutual exclusivity of cell fate options [20, 39].

The self-organized critical-like state of an exploratory ESC regulatory circuit may comprise multiple propagating gene expression cascades that tend to reinforce and support each other if they drive the same cell fate, but cross-antagonize each other if they drive opposing cell fates (for example, pluripotency versus differentiation). The Exploratory hypothesis proposes that it is the ‘interference pattern’ generated by this complex interplay that governs stem cell behaviour and computes cell fate choice.

The term ‘interference pattern’ is specifically chosen to emphasize the similarity between the hypothesized circuitry of ESCs undergoing exploratory cell fate computation and the wave properties of light, which produces interference patterns when two or more light sources of similar frequency interact. In the context of gene regulatory networks, the term ‘interference pattern’ is invoked to underline the crucial importance of multiple parallel gene expression cascades that interact both constructively and destructively to produce an interference pattern that is predicted to give rise to relevant circuitry. Here, we aim to model this emerging circuitry and the associated interference patterns predicted to underpin cell fate computation.

#### Critical-like self-organization

Halley and Winkler propose that critical-like self-organization gives rise to self-regulatory networks [5]. They define critical-like self-organization (also called rapid self-organized criticality) as an interplay between supercritical branching and limited environments. This definition also proposes that there is a fundamental relationship between self-organization and natural selection in evolution: they are two aspects of the same process.

Critical-like self-organization provides a starting point for a general theory of cell fate computation; cells must continually make complex decisions and trade-offs in order to survive. The circuitry of pluripotency must make similar cell fate decisions every time an embryo develops. Hence, the outcome of the decision-making process must be repeatable and robust. So we propose that pluripotent circuitry is likely to undergo ‘graceful failure’, collapsing in a pre-defined and regular manner to achieve accurate cell fate computation in some way, side-stepping the speed-accuracy trade-off.

#### Branching processes

An example of a branching process is the propagation of surnames within a family over time. The process is described by a population of individuals each of which has a branching parameter *m*, for example, the number of children. If the branching parameter is below the critical value necessary for a sustained branching process, *m* < *m*_crit_, then the process will dissipate (the surname will die out). Above the critical value, *m*_crit_ < *m*, the process is termed supercritical. At the critical value of the branching parameter, the branching process is just sustained. See [30] for more detail.

### CellBranch: Modelling regulatory network dynamics with branching process theory

Our principal aim is to capture and integrate the results of multiple high-throughput experiments using a logical and transparent computational framework. This would allow us to model protein expression, particularly TF expression, across multiple layers of stem cell regulation. However, this first requires a sound theoretical framework to understand and predict how regulatory layers self-organize and interact, both within individual cells and between multiple cell types within larger cell assemblies.

Here, we begin to address the problem by describing a novel computational concept derived from the hypothesis of exploratory behaviour described (see Exploratory hypothesis of stem cell fate computation, above) and from branching process theory (see Branching Processes, above).

The utility of such a computational approach relies on the scale-invariant nature of the reproducing units. By its very nature, critical dynamics describes multiple parallel processes that propagate in some way, but because these processes occur within a bounded system, not all can realize their full potential to propagate. Unlike self-organized critical systems, which typically incorporate local stability thresholds, the propagation of a branching process in a critical-like system depends on other processes propagating at that time (although of course there may also be local stability thresholds involved).

The key point is that the critical-like state is achieved or computed via direct interaction among branching processes. In contrast, in self-organized critical systems all of the branching processes are temporally separated such that they cannot interact directly, only indirectly via local stability thresholds.

In our model, branching processes are used conceptually to define boundaries of information flow. ChIP-Seq data are used to capture the entire genome of a pluripotent stem cell, where the expression of each pluripotency TF is defined in terms of a branching process that propagates through time, interacting and competing with others. In this view, fluctuation in the expression of pluripotency genes when mouse ESC are withdrawn from self-renewal conditions (2i) is not trivial: it is central to model dynamics. They are expected to determine differentiation trajectories.

When we consider the expression of a single TF as a distinct branching process, the population of TF molecules can be thought of as the backbone of the branching process as it propagates through time. This can be likened to propagation of a family name through the male offspring; female offspring traditionally fail to propagate the family surname, similar to bursts of TF expression that activate target genes that do not feed back into the transcriptional network. In the context of TF expression, the branching process effectively defines how the population of TF molecules reproduces via the entire regulatory network.

In this sense, the trajectories of individual ESCs are intrinsically knowable and able to be calculated from patterns of competition and interference among cascades of gene expression bounded by the cistrome of each TF. Genome location data, which describe interactions between TFs and other genes at genome-wide scales, can be used to simulate these branching processes and estimate patterns of interference that give rise to individual cell trajectories.

#### The CellBranch simulation model

Here, we present our new computational model of regulatory network dynamics, applied to mouse ESCs.

Our primary concern here is to capture the self-organization of cellular circuitry such that the trajectories of individual cells can be better understood, and ultimately, directed. We anticipate that both the shattering core pluripotent circuitry together with its re-ignition can be defined within a single computational algorithm. Here we begin this process.

We model a single cell, not a population of cells. We justify this decision on the grounds that cells have internal decision making, even though they may be influenced by their environment. As a consequence of our choice of model system—a single ESC—we are not able to model decision making of populations of cells at this stage.

Although the significance of external signals is clear (demonstrated unambiguously with the 2i system), we here ignore external factors in order to focus on the intrinsic ability of regulatory networks to self-organize in their absence. To understand the role of external factors in self-organizing systems, it is important first to understand systems without them. Debate between intrinsic versus extrinsic control of stem cell fate decisions has been ongoing for decades and is again emphasized in a recent model of pluripotency network decisions [8].

We utilise ChIP-Seq data from ESC cultured in serum plus LIF conditions, arranged into a square grid of 50kb gene segments (supplied by co-authors Smith and Dietmann). On this grid, we superpose a branching process in which only gene segments with one or more binding sites for a given TF, X, are able to be activated and so transcribe further molecules of X, thus sustaining the branching process. If a gene segment is activated at a given timestep, it produces *m* units of TF, and becomes deactivated; if a TF binds to a gene segment, the segment becomes activated. We allow for constructive communication between cistromes to investigate the effect that one branching process can have on others in the same cell.

Our detailed model is described in the final Model section below. The reader is also referred to Halley’s publications on the Transcription Factor Branching Process (TFBP) [2, 3, 5]. The CoSMoS workshop paper of Greaves et al. [30] provides details of the original single-cistrome simulation; the simulation experiments presented in that work serve principally as calibrations of the simulation software. Here we present the multiple communicating cistrome results, demonstrating how the model readily generalises to layering additional cistromes, thereby having tamed some of the complexity.

In summary, our TFBP model assumes that a TF producing segment in cistrome X produces the TF of cistrome X. In the case of coupled cistromes, this TF may be ‘transferred’ to a different cistrome; that is, if cistromes X and Y are coupled, a TF producing segment in cistrome X may probabilistically produce the TF for cistrome Y. These TFs need not be *directly* produced; there may be a cascade of production, but the TFBP model collapses this cascade. This abstraction from the detailed biology allows us to model the highly complex process dynamics in a relatively simple manner. We use experimental cistrome data (number of TF producing segments, number of coupled segments) to parameterise the simulations derived from this model. For more details, see the “Model” section below.

## Results

### Existing findings for single cistromes

From the simulation of the TFBP, we can demonstrate the existence of m_crit_ values, values of the branching parameter below which the simulation rapidly dissipates and above which supercritical branching can take place; see Greaves et al [30] for details.

The model includes the following parameters and values:

- *p*: the total number of gene segments that contain a binding site for a given TF, for example Nanog, and which are therefore in the Nanog cistrome
- *r*: the number of segments in the cistrome that can be transcribed into TF products (that can be ‘active’)
- *m*: the branching parameter for the cistrome: the number of TFs that an activated segment emits in a timestep
- *s*_*t*_: the number of segments that are activated (being transcribed) at time step *t*
- *σ*: the number of segments that can be activated that are shared between cistromes

**Fig 1:**
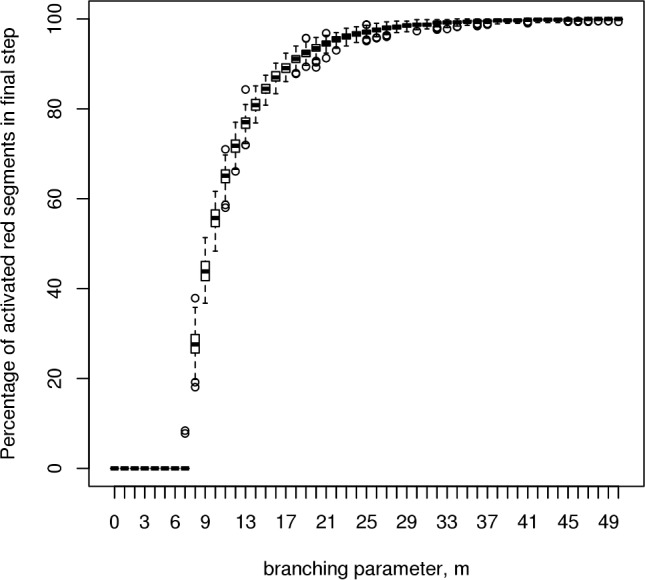
Nanog cistrome critical branching value. This simulation experiment illustrates the existence of a critical value for the branching ratio, *m*, in a single cistrome system. For the Nanog cistrome data used in generating our model, this critical value is found to be 8. Below this value of the branching ratio, all simulation runs dissipate within the first 200 timesteps, whereas above this value, an increasing number of simulations achieve sustainable branching processes (200 runs, each measured over 1000 timesteps).

The determination of m_crit_ for Nanog, Sox2 and Oct4 is found in Greaves et al [30]. (The experiments were repeated with the new multi-cistrome code used in this paper, run with a single cistrome; the same results were found.) The determination of m_crit_ for Nanog is illustrated in Fig 1. The value of m_crit_ for the Nanog cistrome (8) is found to be somewhat higher than that for the other core pluripotency cistromes of Oct4 and Sox2 (6 and 7 respectively). The observed m_crit_ results are summarised in the final table below.

An infinite limit model discussed in [30] is used to calculate an estimate of m_crit_; in this limit the system tips from fully dissipated to supercritical immediately. Fig 1 is not such a step function. The finite sized, noisy, system tips sharply from dissipated to ignited at the experimentally observed value m_crit_, but then requires a somewhat higher value of m to become fully saturated. The observed m_crit_ is slightly higher than the infinite limit value (Table 1), due to noise. Importantly, even though the system is noisy, it still maintains supercriticality in the face of fluctuations in all runs with m_crit_ ≤ m.

**Table 1:** The values of the key simulation parameters.

parameter	Nanog	Sox2	Oct4	cMyc
*p*	4316	3330	2540	2961
*r*	631	542	466	864
*p*/*r*	6.8	6.1	5.5	3.4
*m*_crit_	8	7	6	4

**Fig 2:**
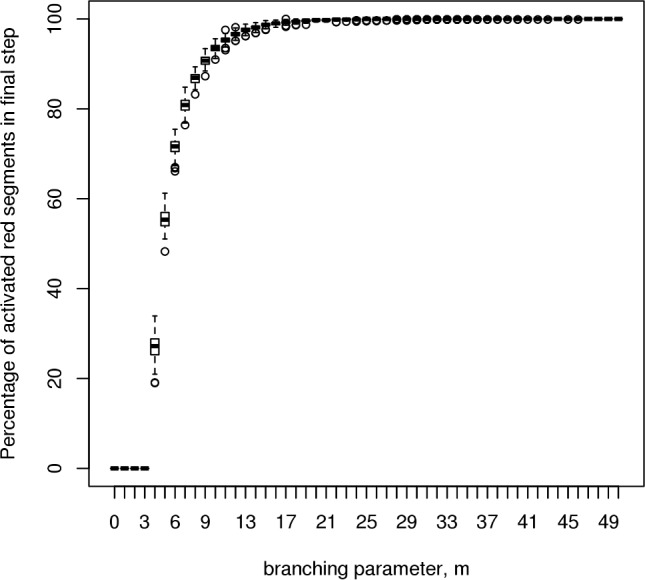
cMyc cistrome critical branching value. This simulation experiment illustrates the existence of a critical value for the branching ratio, *m*, in a single cistrome system. For the c-Myc cistrome data used in generating our model, this critical value is found to be 4. Below this value of the branching ratio, all simulation runs dissipate within the first 200 steps, whereas above this value, an increasing number of simulations achieve sustainable branching processes (200 runs, each measured over 1000 timesteps).

Parameters *p*, *r* (determined from experimental data) and the measured *m*_*crit*_, (determined from simulations) for the four cistromes studied in this work. The parameters are defined in the text (see above). For measured Nanog, Oct4 and Sox2 values of *m*_*crit*_ see Greaves et al. [30]. For measured cMyc value of *m*_*crit*_ see Fig 2.

We have also verified that that m_crit_ is not affected by whether the initial value s_0_ = *r* or s_0_ = 0.5*r* and that results are scalable i.e. we can alter the values of *p* and *r* in a cistrome, providing that the ratio *p* / *r* remains constant and obtain the same results [30].

In addition to these previously existing findings, we have run similar simulations for the cMyc cistrome to determine its value of m_crit_ (see Fig 2 and Table 1). This cistrome has a relatively low value of m_crit_ (of 4) due to its relatively high number of active sites compared to the other cistromes investigated.

### Coupling cistromes can drive to criticality

**Fig 3:**
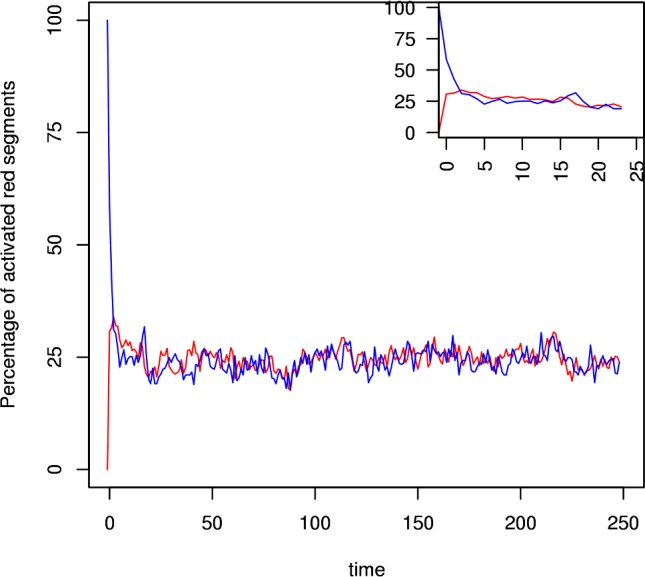
Oct4 igniting and driving a fully dissipated Nanog cistrome to criticality. A critically branching Oct4 cistrome (blue trace) with initial activation s_0_ = r and m = m_crit_ at time t = 0 drives the branching in an initially fully dissipated Nanog cistrome (red trace) with initial activation s_0_ = 0 and m = m_crit_.

**Fig 4:**
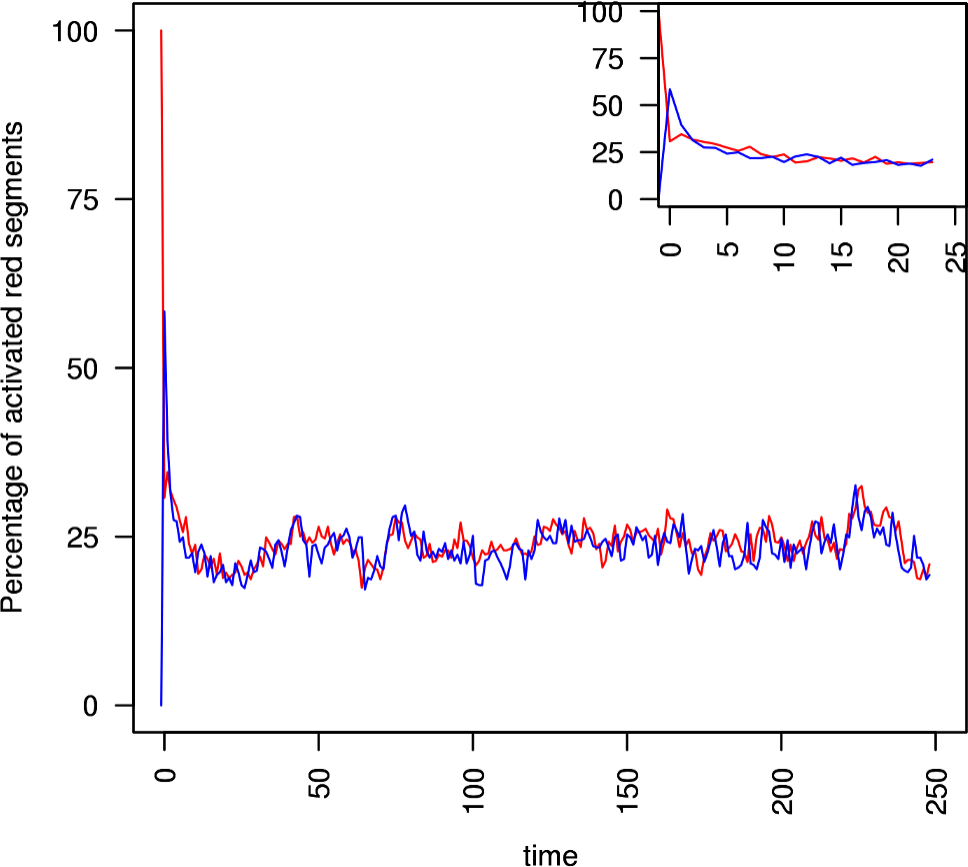
Nanog igniting and driving a fully dissipated Oct4 cistrome to criticality. A critically branching Nanog cistrome (red trace) with initial activation s_0_ = r and m = m_crit_ at time t = 0 drives the branching in an initially fully dissipated Oct4 cistrome (blue trace) with initial activation s_0_ = 0 and m = m_crit_.

The multi-cistrome simulation shows that one active cistrome can “ignite” a fully dissipated cistrome, and drive it to criticality. This is illustrated in two cases. The critically branching Oct4 cistrome can drive fully dissipated Nanog cistrome to criticality (Fig 3). The figure shows the first of 200 simulation runs carried out with these initial conditions (the others runs are qualitatively similar). The inset shows the first 25 timesteps. Similarly, the critically branching Nanog cistrome can drive a fully dissipated Oct4 cistrome to criticality (Fig 4). These show in each that, although the cistrome begins the simulation fully dissipated, it is swiftly ignited to sustainable branching by the critically branching cistrome to which it is coupled.

This is a non-trivial result because: (i) the driving cistrome is working just at the critical value of *m* needed to sustain itself; (ii) the driving cistrome incurs a “cost” to do so, because some of the TPs it produces are transferred to the other initially dissipated cistrome, rather than being used to maintain its own activity.

### Coupling cistromes can reduce m_crit_

In addition to igniting a dissipated cistrome, an effect of coupling cistromes is the reduction of m_crit_.

In [30] we use an infinite limit model to calculate an estimate of m_crit_ in a single cistrome. We here use a similar approach to calculate an estimate in the reduction of m_crit_ in coupled cistromes. This infinite limit case is essentially noiseless, with each TF site being ignited the average number of times.

Consider the case of two cistromes, *X* and *Y*. At time *t* let there be *s*_*t*_^X^ sites active in cistrome *X*. In the TFBP model, each of these active sites emits *m*^X^ TFs, so a total of *s*_*t*_^X^ × *m*^X^ TFs are emitted. Let each of these TFs be absorbed by a separate site with uniform probability. There are three cases (three kinds of sites):

1. Activate a Y cistrome (shared) site. A fraction σ / *p*^*X*^ of the sites are shared, and the model assumes that a TF absorbed by one of these passes to the Y cistrome. So *s*_*t*_^*X*^ *m*^*X*^ σ / *p*^*X*^ TFs in total are passed to the Y cistrome, activating that number of sites in the Y cistrome in the next timestep.
2. Activate an X cistrome site. A fraction (*r*^*X*^– σ) / *p*^*X*^ of the sites can be activated in the *X* cistrome, and a TF absorbed by such a site activates it in the next timestep. So *s*_*t*_^*X*^ *m*^*X*^ (*r*^*X*^– σ) / *p*^*X*^ sites in the X cistrome are activated by these TFs
3. Absorbed. The remaining fraction of sites absorb a TF and do not produce a TF in the next timestep.

Similar arguments, *mutatis mutandis*, hold for TFs emitted by cistrome Y. So at timestep *t*+1, the number of active sites in cistrome *X* is those activated from cistrome *Y* plus those activated from cistrome *X*

$$s_{t+1}^{X}=\frac{s_{t}^{X}m^{X}(r^{X}-\sigma )}{p^{X}}+\frac{s_{t}^{Y}m^{Y}\sigma }{p^{Y}}$$

A similar equation holds for *s*_*t*+1_^*Y*^. At the critical tipping point, *s*_*t*+1_ = *s*_*t*_. We take these two equations, eliminate *s*^*X*^ / *s*^*Y*^, then solve for *m*^*X*^, to get

$$m^{X}=\frac{p^{X}(p^{Y}-m^{Y}r^{Y}+m^{Y}\sigma )}{(p^{Y}-m^{Y}r^{Y})(r^{X}-\sigma )+m^{Y}r^{X}\sigma }$$

This gives the infinite limit predicted value of *m*^*X*^ in the case of a given *m*^*Y*^. If we substitute *m*^*Y*^ = *p*^*Y*^/*r*^*Y*^, the infinite limit single cistrome critical value for *Y*, we get *m*^*X*^ = *p*^*X*^/*r*^*X*^. That is, in the infinite limit, the critical values are unchanged. Alternatively, if we substitute *σ* = 0 (isolated cistromes), we also recover the original predicted value of *m*^*X*^. However, if we substitute the *observed* critical value in the finite sized noisy case for *m*^*Y*^, and the *experimentally derived* value of σ (shown in Table 2) we get a different prediction for *m*^*X*^, as shown in Table 3.

**Table 2:** Cistrome overlap.

	Sox2	Oct4	cMyc
Nanog	287	194	265
Sox2		237	252
Oct4			268

The experimentally determined values of σ, the degree of cistrome overlap, in terms of the number of gene segments that can be transcribed to TF products that are shared between two cistromes.

**Table 3:** The predicted change in critical branching factor in coupled cistromes.

		cistrome X *p*/*r*	Nanog 6.8	Sox2 6.1	Oct4 5.5	cMyc 3.4
cistrome Y	*p*/*r*	mcrit	8	7	6	4
Nanog	6.8	8	–	4.9	4.0	2.9
Sox2	6.1	7	6.0	–	4.6	3.1
Oct4	5.5	6	6.3	5.6	–	3.2
cMyc	3.4	4	5.0	4.2	3.6	–

Cistrome Y is held at its observed critical value mcrit; the predicted value of cistrome X’s m_crit_ when the cistromes are coupled is lower than its value when cistrome X evolves in isolation.

Table 3 shows the prediction that critical value for cistrome X should fall when coupled with cistrome Y run at its observed critical value. Under the assumptions used to generate Table 3, cistrome Y is being run at a higher rate than it needs *in the infinite limit*, and the excess TF production can be used to lower cistrome X’s required rate. The question is: does this reduction carry over in the finite case, or does the presence of noise, requiring a higher than predicted rate to sustain, negate any such change? Our simulation results, for several of these cases, are presented below.

**Fig 5:**
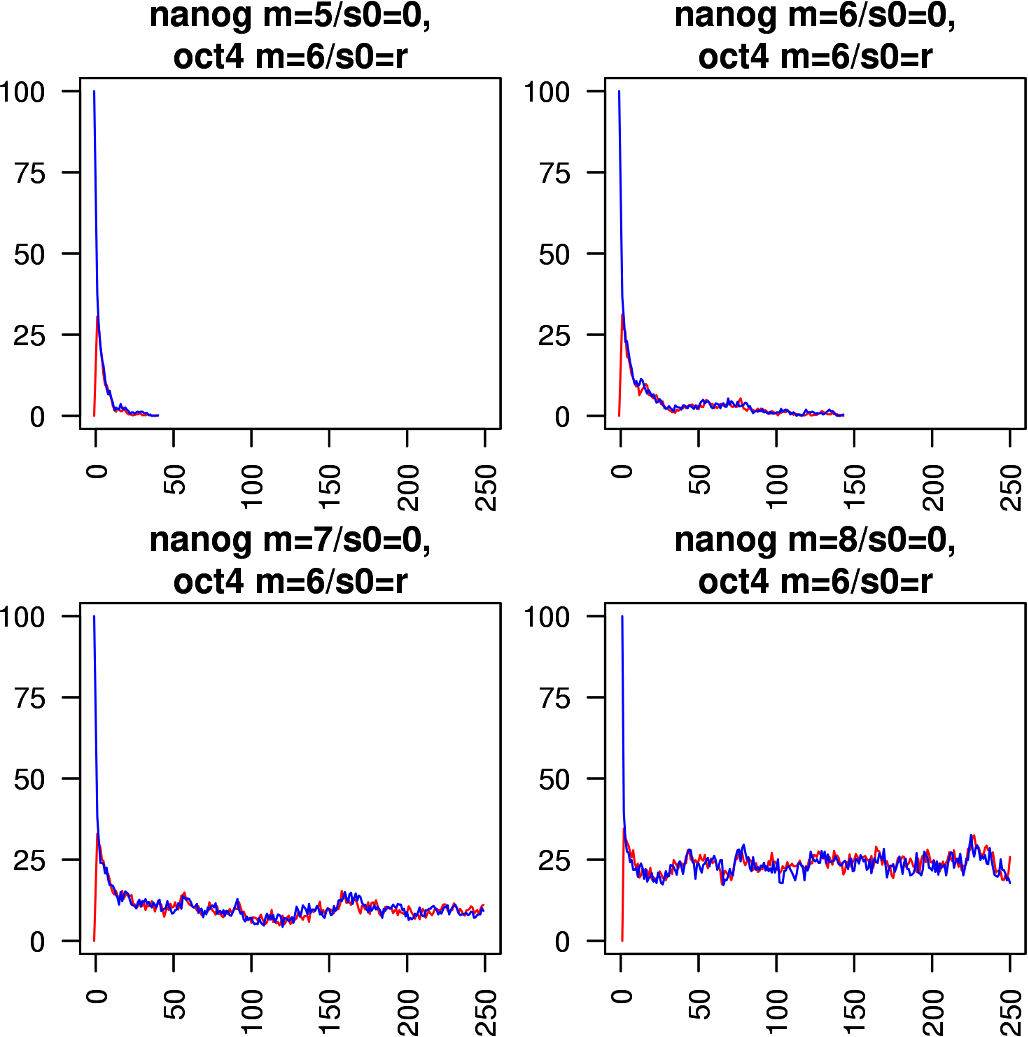
Coupling the Nanog cistrome to the Oct4 cistrome. The effect of coupling a subcritically or critically branching Nanog cistrome (red trace) to a critically branching Oct4 cistrome (blue trace). The m_crit_ value in the Nanog cistrome is lowered from 8 to 7.

**Fig 6:**
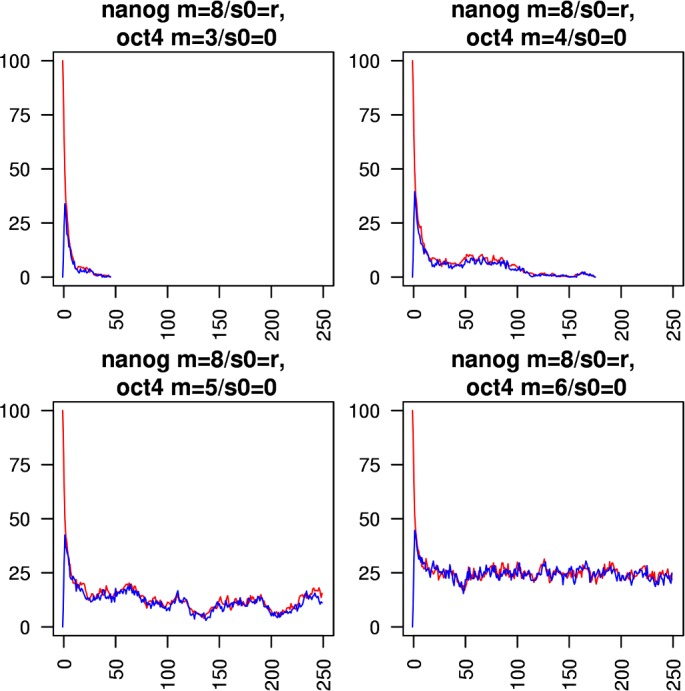
Coupling the Oct4 cistrome to the Nanog cistrome. The effect of coupling a subcritically or critically branching Oct4 cistrome (blue trace) to a critically branching Nanog cistrome (red trace). The m_crit_ value in the Oct4 cistrome is lowered from 6 to 5.

**Fig 7:**
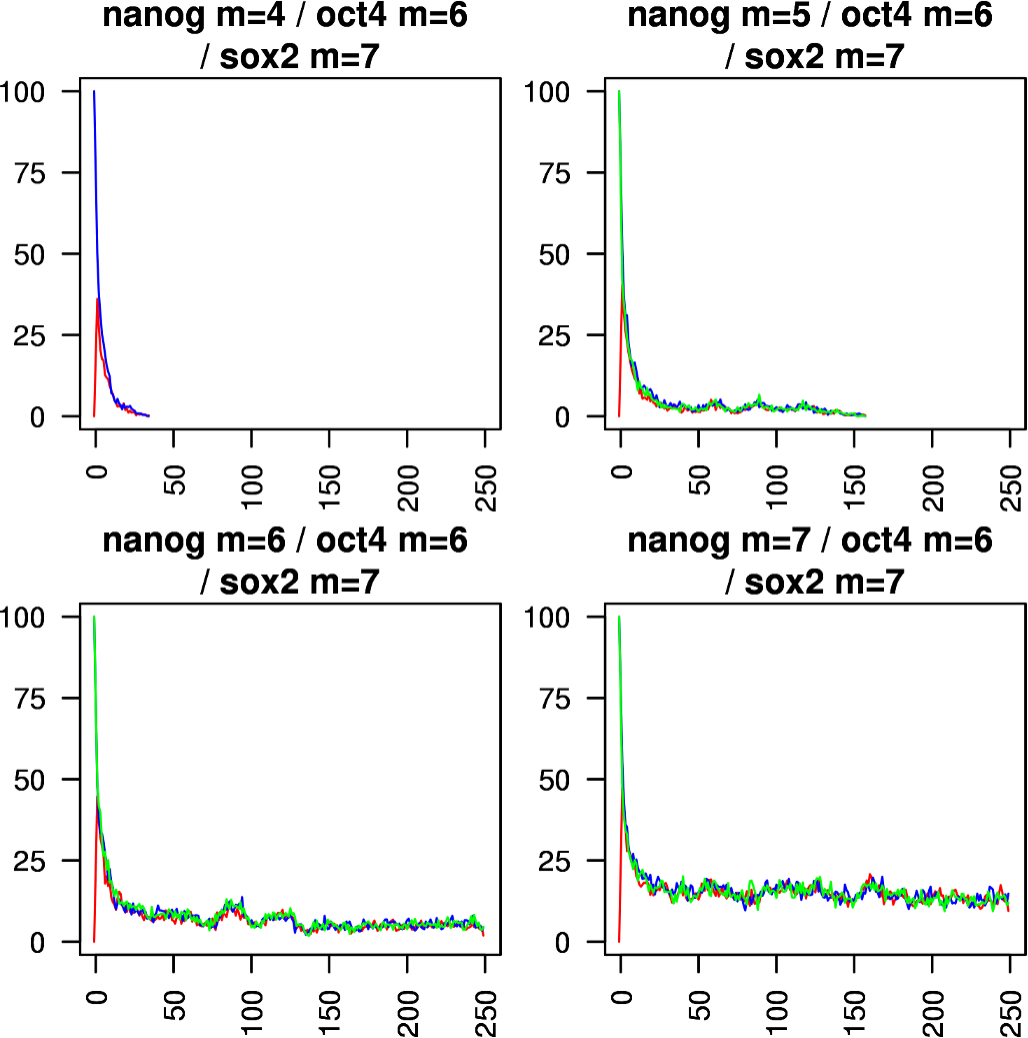
Coupling the Nanog cistrome to both the Oct4 and Sox2 cistromes. The effect of coupling a subcritically or critically branching Nanog cistrome (red trace) to a critically branching Oct4 cistrome (blue trace) and a critically branching Sox2 cistrome (green trace). The m_crit_ value in the Nanog cistrome is lowered from 8 to 6.

**Fig 8:**
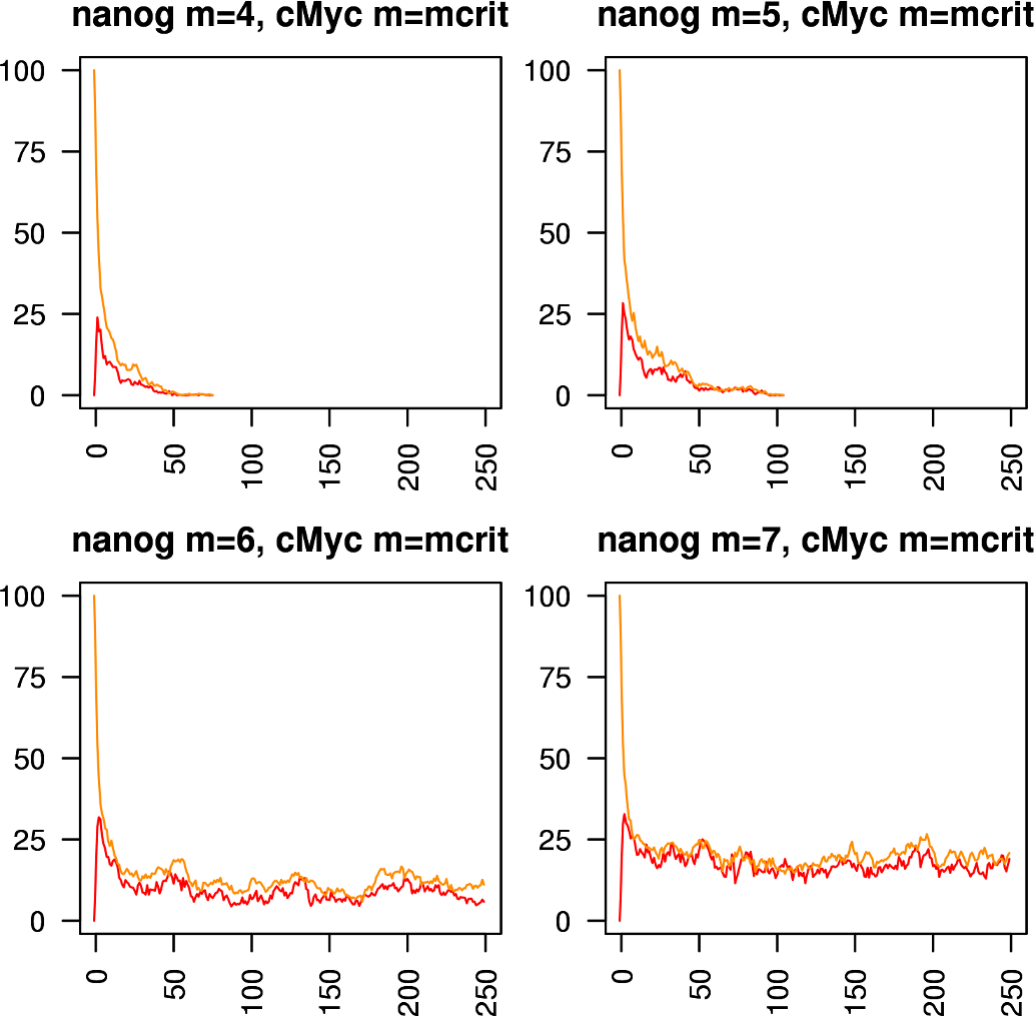
Coupling the Nanog cistrome to the cMyc cistrome. The effect of coupling a subcritically or critically branching Nanog cistrome (red trace) to a critically branching cMyc cistrome (orange trace). The m_crit_ value in the Nanog cistrome is lowered from 8 to 6.

**Fig 9:**
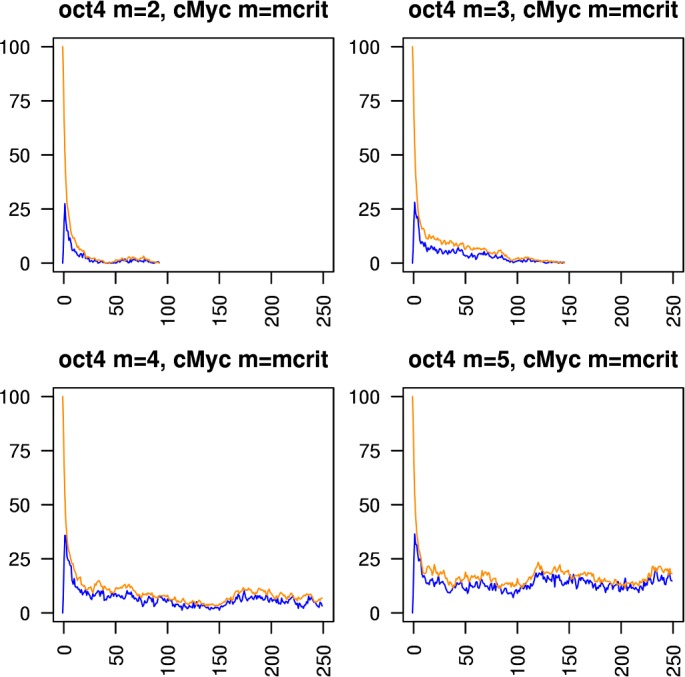
Coupling the Oct4 cistrome to the cMyc cistrome. The effect of coupling a subcritically or critically branching Oct4 cistrome (blue trace) to a critically branching cMyc cistrome (orange trace). The m_crit_ value in the Oct4 cistrome is lowered from 6 to 4.

**Fig 10:**
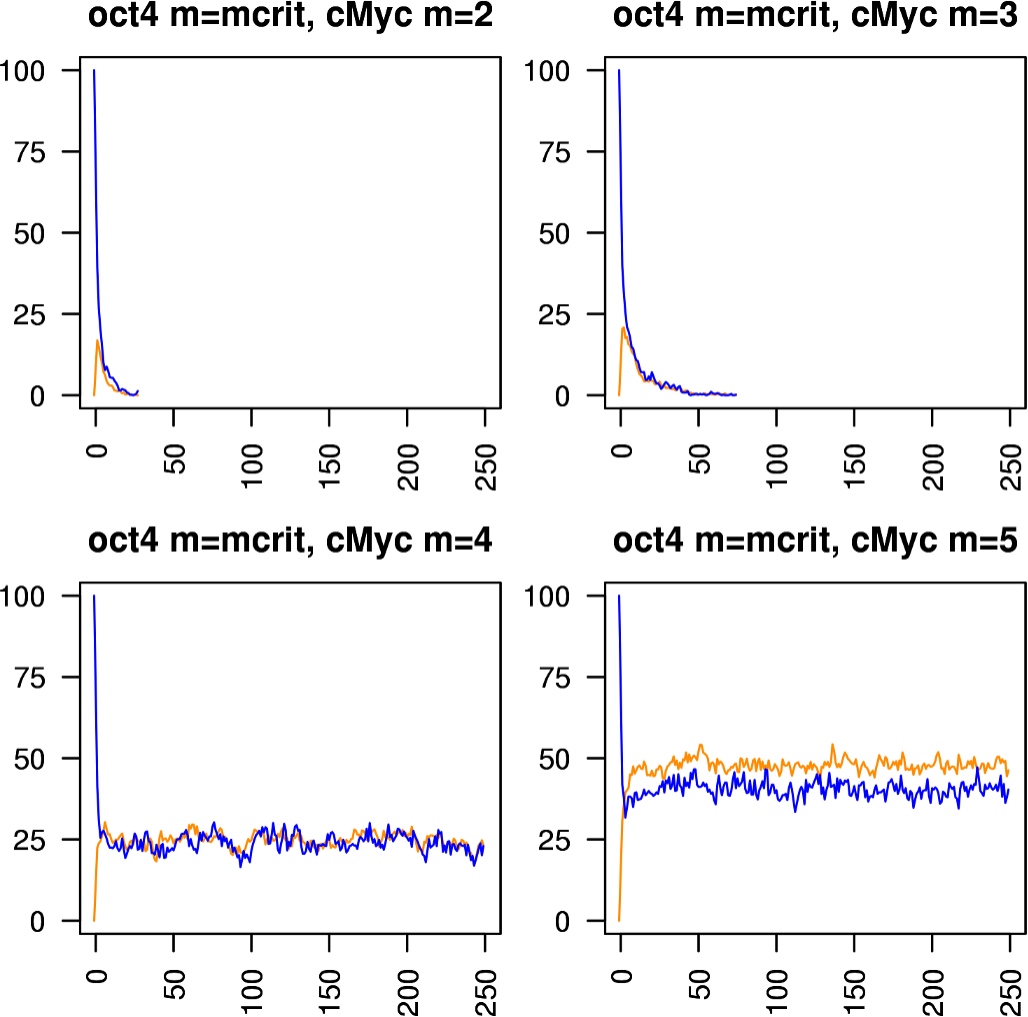
Coupling the cMyc cistrome to the Oct4 cistrome. The effect of coupling a subcritically or critically branching cMyc cistrome (orange trace) to a critically branching Oct4 cistrome (blue trace). The m_crit_ value in the cMyc cistrome is unchanged at 4.

Figs 5–10 show the behaviours of various combinations of coupled cistromes. Each plot shows the first 250 of 1000 timesteps performed, and shows the first run from a set of 200 runs performed; the others runs are similar. In each case, the critically branching cistrome(s) can drive the branching process in the other cistrome, and the sub-critically branching cistrome dissipates the branching process in the critically branching cistrome(s). Coupling the Nanog cistrome to that of Oct4 (with Oct4 having its *m* value fixed at its observed value m_crit_ = 6) reduces m_crit_ for the Nanog cistrome by one (Fig 5). Similar results hold for coupling the Nanog cistrome to Sox2. Coupling the Oct4 cistrome to that of Nanog (with Nanog having its *m* value fixed at its m_crit_ = 8) reduces m_crit_ for the Oct4 cistrome by one (Fig 6). Coupling the Nanog cistrome to both the Oct4 and the Sox2 cistromes reduces m_crit_ for the Nanog cistrome by 2 (Fig 7). The c-Myc cistrome is extensively overlapped with the core pluripotency cistromes. Coupling the Nanog cistrome to the c-Myc cistrome alone reduces m_crit_ for the Nanog cistrome by 2 (Fig 8). Hence the c-Myc cistrome has twice the effect on Nanog m_crit_ as either Oct4 or Sox2 alone. Similar results hold for Oct4. Coupling the Oct4 cistrome to that of cMyc cistrome reduces the value of m_crit_ for the Oct4 cistrome from 6 to 4 (Fig 9). Coupling the cMyc cistrome to that of Oct4 cistrome leaves the value of m_crit_ for the cMyc cistrome unchanged at 4 (Fig 10).

The various reductions in m_crit_ caused by coupling cistromes are summarised in Table 4. Similar to the single cistrome case, the observed values of m_crit_ are a little higher than the infinite case predictions. In all cases but one the observed coupled value is nevertheless lower than the observed uncoupled value, demonstrating that the reduction can be maintained in the presence of noise. In the case of Oct4 driving cMyc, there is no reduction in the observed value of m_crit_, possibly because the value is so low in the first place, and so any reduction would be proportionally smaller.

**Table 4:** The effect of coupling on a cistrome’s values of m_crit_.

cistrome Y	cistrome X m_crit_	Nanog 8	Sox2 7	Oct4 6	cMyc 4
Nanog	8	–	4.9	4.0 / **5**	2.9
Sox2	7	6.0 / **7**	–	4.6	3.1
Oct4	6	6.3 / **7**	5.6	–	3.2 / **4**
cMyc	4	5.0 / **6**	4.2	3.6 / **4**	–
Sox2+Oct4	7+6	**6**			–

Normal text shows the predicted infinite limit predictions (reproduced from Table 3 for comparison); bold text shows the measured values for m_crit_ for various couplings. The second cistrome *Y* is run at its original value of m_crit_. Sox2 and Oct4 can each decrease Nanog’s m_crit_ by 1, and working together by 2. cMyc on its own can reduce Nanog’s m_crit_ by 2. Nanog can reduce Oct4’s m_crit_ by 1, cMyc can reduce it by 2. Oct4 does not, however, reduce cMyc’s m_crit_.

## Discussion

Our simulation model supports the argument that critically branching processes in cistromes can drive sub-critically branching processes in other cistromes to criticality, as described by the branching process model proposed by Halley and Winkler [5].

Our simulation of the theoretical TFBP occurring within the various TF cistromes of the genome clearly illustrates that there is a critical value of the branching ratio below which branching effectively ceases to occur. Critical or super-critical branching, however, can lead to a sustained branching process in a given cistrome. This is roughly equivalent to saying that a certain minimum transcription of genes for a given TF cascade is required for its sustained production throughout time.

Our simulation model also illustrates the interplay between branching processes in cistromes, in that critically branching processes in one cistrome can drive the processes in other cistromes to which they are coupled, and can also lower the critical value of the branching parameter in those cistromes by one (Oct4 and Nanog, Sox2 and Nanog, Oct4 and Sox2) or by two (cMyc and Nanog, cMyc and Oct4, cMyc and Sox2, Sox2 with Oct4 acting on Nanog).

The simulation model as it stands provides evidence that the Transcription Factor Branching Process (TFBP) of Halley and Winkler [5] has utility in describing the regulation of TF gene regulatory networks. This is a first step towards the understanding of the non-equilibrium dynamics of the core pluripotent network of ESCs. The simulation additionally provides some low level description of the interaction between TF cistromes, demonstrating that igniting transcription of one TF can further prompt the ignition of transcription of other TFs. To gain a more realistic insight to the extent of this effect we need to, ideally, extend the model to incorporate fully interacting branching processes and also to allow for inhibitory binding of TF to sites in the cistrome of TFs whose transcription they repress.

### Conclusions and further work

We have taken a first step towards the creation of a multi-layered model of the stem cell regulatory network and in our opinion, these results are interesting and begin to tease out the utility of the Branching Process Theory in understanding Stem Cell fate computation. We have demonstrated that a regulatory network may self-organise from one equilibrium state to another, through ignition of a coupled dissipated cistrome. Understanding the complex dynamics of such self-organising changes of state possible with communicating cistromes may ultimately give insight into how a pluripotent state can tip to a differentiated state, and may help in understanding how to control and even reverse such tipping.

However, our model does not, as yet, capture all the biologically-relevant dynamics. For example, in our current model, a TF can interact with genes in another cistrome only in a stimulatory manner. We need to include the possibility of inhibitory TF binding of TFs to their binding sites on the various gene segments in the cistromes modelled. We therefore need to develop a Domain Specific Language to allow a standardised way of describing the TF circuitry (the pattern of excitatory and inhibitory relationships between individual TFs) to be modelled in the simulation.

The current model is essentially ‘blind’ to the identity of any TFs produced via transcription of the gene segments in any given cistrome, and also ignores the potential requirement for multiple TF to bind to a gene segment in order to activate it (or inhibit it). There are also other considerations that we need to take on board in order to capture further biologically-relevant dynamics. We also need to accommodate combinatorial binding of TFs to gene segment promoter sequences, as a gene may require binding of specific combinations of TFs to their binding sites in order to promote transcription of the gene segment. Such combinatorial TF binding to enhancer sites can impart transcriptional synergy in a future multicellular model.

We need to include expression data to factor in the strength of activation: currently each gene segment is assumed to produce the same amount of TF each timestep. We also need to include some concept of TF half-life into the model as currently all TFs are assumed to survive and remain bound to their binding sites for a single simulation time-step only. As epigenetic histone modifications may help to shape the circuitry of self-organization, it would also be useful to be able to take into account epigenetic considerations and the effect of enhancer sites within the cistromes modelled.

Our model is currently aspatial, in that it lacks any detail of three-dimensional genomic architecture, which affects how TFs access their binding sites. Inclusion of histone data to incorporate spatial effects is a future goal.

Additionally, we remain uncertain of how the degree of cistrome overlap (number of shared segments) determines the extent of the effect of cistrome coupling on reduction of m_crit_. More experimental data on this overlap, particularly in the case of multiple cistromes, is needed to investigate this.

The model presented represents a novel example of self-organization that may apply to other complex systems of interest from a theoretical point of view because it helps to demonstrate how distributed interactions among units result in higher order emergent behaviours. Such complexity could provide dynamic templates of organization upon which natural selection builds additional elaborations [5].

However, even this extremely pared down implementation of the TFBP demonstrates the ability of coupled TFBPs (equivalent to transcription patterns) to influence and modulate each other’s branching behaviour via constructive interference as suggested by the theoretical model proposed by Halley and Winkler [5].

The code for the simulation, batch scripts for running the simulation on an SGE enabled compute cluster, Python scripts for generating real or synthetic cistromes, and example R scripts for processing simulation results into graphical form, are all available on GitHUB at: github.com/CellBranch/CellBranch

## Model

### Original single cistrome model

**Fig 11:**
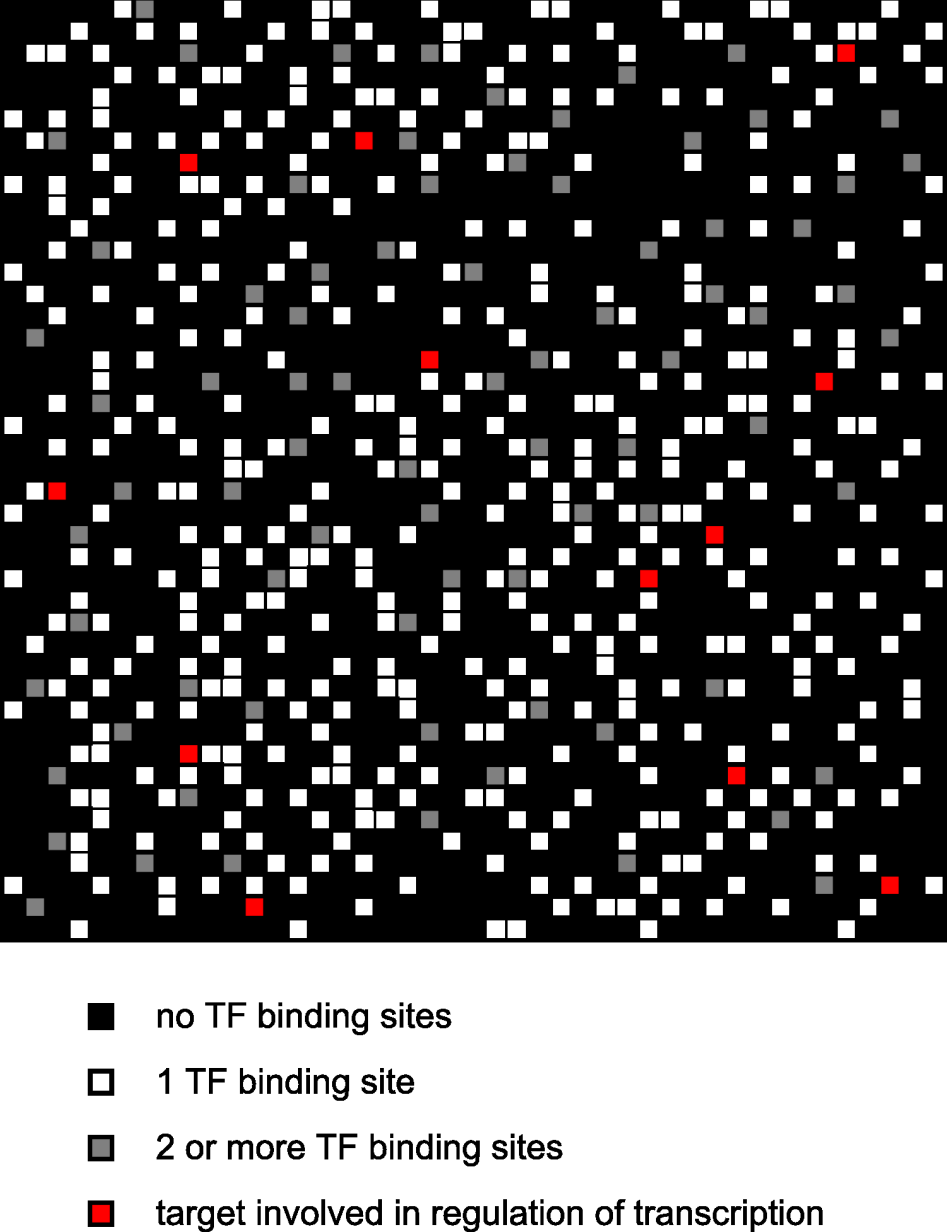
A representation of a set of ChIP-Seq data for a cistrome. Red squares represent 100kB segments of DNA that contain at least one binding site for a TF. The white and grey squares belong to the cistrome but encode products that are not themselves TFs. Black squares are 100kB segments of DNA that do not belong to the cistrome. From [30].

**Fig 12:**
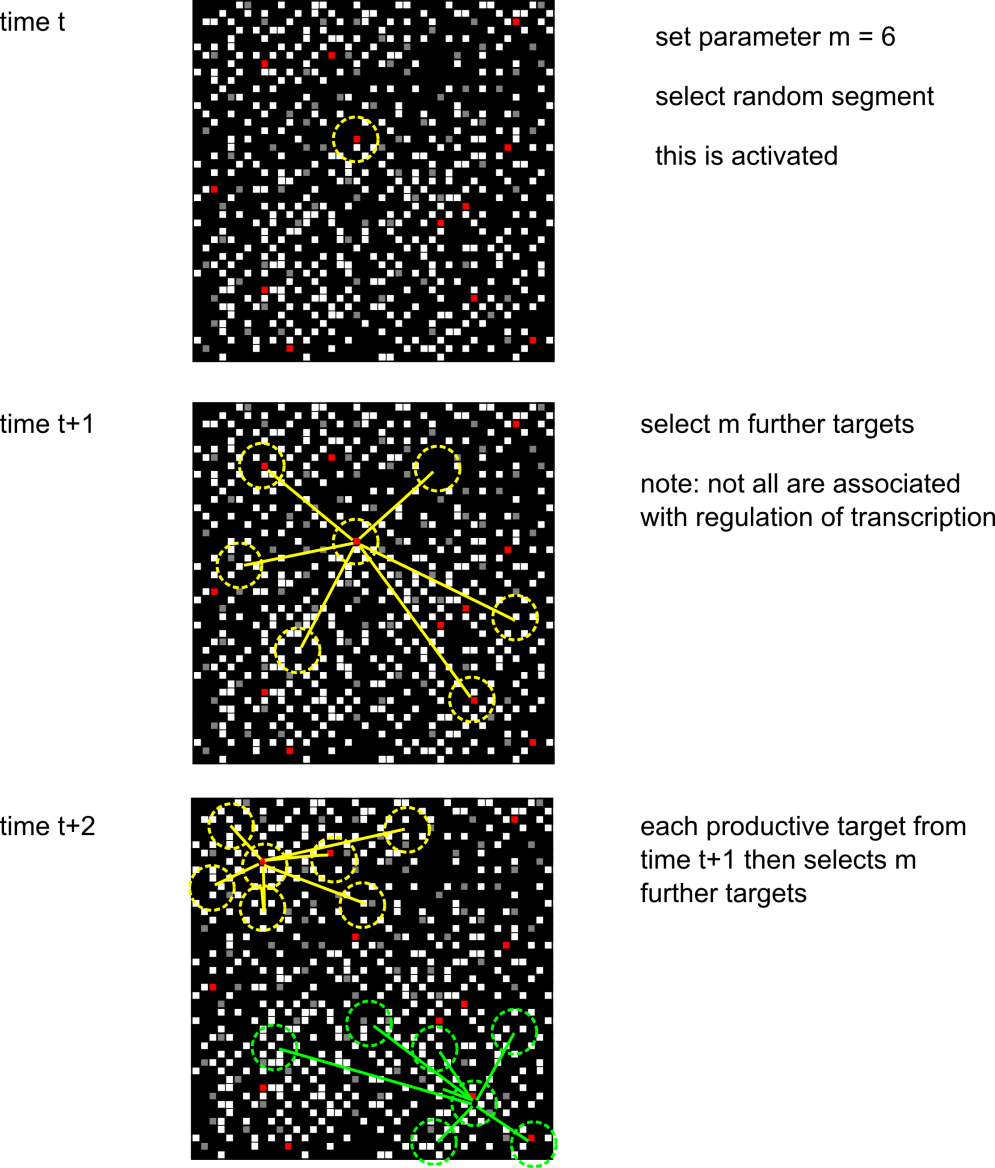
A branching process on ChIP-Seq data cistrome. A branching process representation of the overall flow of regulatory information, which serves as the basis of our earlier single cistrome simulation. From [30].

In Greaves et al. [30] we detail the development of a simulation of a single cistrome branching process using the iterative CoSMoS simulation design protocol [40], taking the Transcription Factor Branching Process (TFBP) Model [2, 3, 5] as our initial domain model. That simulation was written as object-oriented code in Java, using the MASON simulation development environment. The reader is referred to Greaves et al. [30] for the relevant design details, and assumptions made about the domain. The context of the simulation development remains the same as in our previous work, i.e. the investigation of the TFBP approach to modelling stem cell fate computation. Fig 11 shows how the ChIP-Seq data is used to produce a model of a single cistrome. Fig 12 shows how this single cistrome is used to underpin the TFBP model.

In Greaves et al. [30] we have reasoned that if the activities of single TFs can be adequately described as a critical-like Branching Processes, as suggested by our results in that publication, then their interplay should define a critical-like genome-wide interference pattern. This pattern would then in some way, capture the nature of the entire pluripotency TF regulatory network [3].

### Multiple communicating cistrome model

We now wish to gain a deeper understanding of the behaviour of constructive interference between interfering branching processes. In particular, we aim to characterise TF branching processes and how they might propagate in the presence of cross-cistrome communication. So we now discuss the extension of the earlier, simple model Greaves et al. [30], to a model of two or more interacting, branching cistromes, to enhance the biological relevance of the simulation. We also need to allow for segments that are shared by multiple cistromes and to specify TF sharing algorithms. These refinements of the simulation require us to make further assumptions about system behaviour and to revise exiting ones. Most obviously, this includes assumptions about how cistromes communicate throughout the simulation. By communication, we mean the transfer of TF branching behaviour products from one cistrome to the appropriate binding sites on another. We assume that the inclusion of constructive interference of TFBPs is a sensible increment in the model of the system, but acknowledge that it does not yet yield a simulation of full biological relevance.

In Greaves et al. [30] we use the language of the most abstract of our models of the system, our ‘sparking posts’ model, but here we use the language of the more biological abstraction, i.e. cistromes and segments and ‘transcription’. In our new multi-cistrome model, the TF binding sites in a cistrome can be occupied by TFs transcribed from genes in the same cistrome or those from another cistrome. We have found it useful to implement the model so that we can distinguish the separate origins of incoming TFs–same cistrome or different cistrome, with those TF incoming from a different cistrome being bound to a binding site that we nicknamed the ‘spark bucket’ in our abstract sparking posts model, but which is merely the equivalent of a second TFBS for the appropriate incoming TF in our abstract biological model. We start our augmentation of the simulation by examining the case of cooperation between two TFs at a genome-wide scale. This is still far from a biologically realistic system as very many TFs will intercommunicate to regulate cellular systems i.e. multiple TFBPs will interfere constructively and destructively such that we would expect to ultimately be able to generate the interference patterns predicted to underpin cell circuitry self-organization.

Strictly speaking, in biology, any segment shared by two or more cistromes will have a TF binding site for each of the TFs produced by the cistromes which share the gene segment (remembering that in our simplified model cistrome X branching gives rise to TF X at all times if a TF product binds to a binding site in cistrome X).

However, since the model allows a segment in cistrome X to produce TF products when we have either a TF derived from cistrome X or a TF derived from any other coupled cistrome, then it is not necessary for us to include the possibility of TF transfer from more than one cistrome to the segment in Cistrome X. Again, we believe that the distinction between this description and the one we actually employ will be subtle, but acknowledge that it should be fully tested before we progress the model further.

We also assume that TFs can remain bound to their binding sites for one simulation time-step only. At each time-step a segment in a particular cistrome, let us call this cistrome X for sake of argument, can receive a TF molecule resulting from branching within Cistrome X. It can also receive a TF molecule generated by branching within another cistrome, say Y, which shares this particular segment with Cistrome X. We have further assumed that in any single time-step, only one TF from another cistrome can be transferred to a corresponding binding site in Cistrome X.

**Fig 13:**
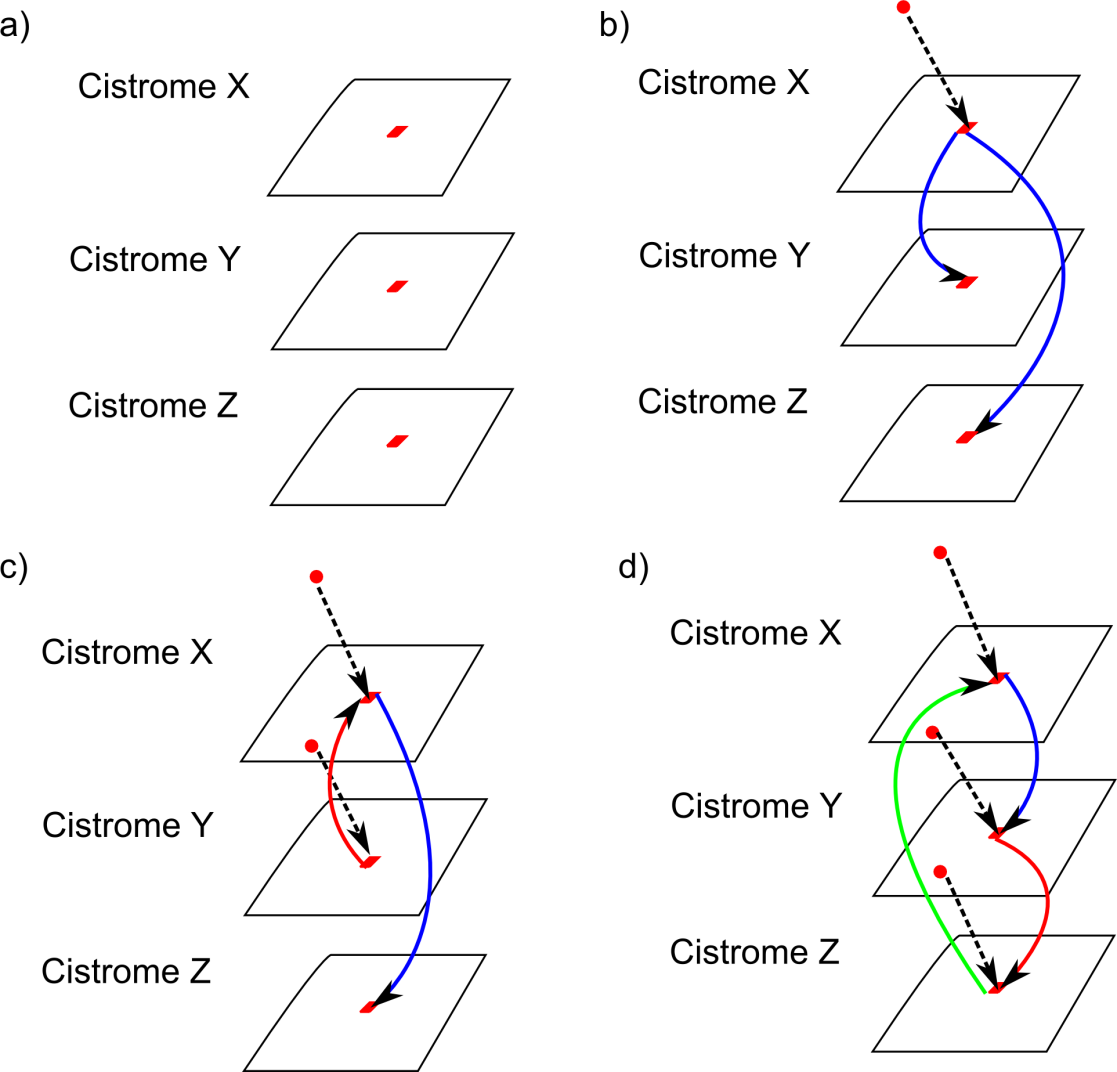
Communication between cistromes. An illustration of communication between cistromes undergoing TFBPs. The details of our assumptions about how inter-cistrome communication works in our model are presented in the main body of the text. Dashed lines represent a TF binding to a segment in a cistrome; solid lines represent transfer between cistromes; blue is the first transfer, red the second, and green the final transfer. (a) three interacting cistromes X, Y, Z that share a gene segment (red square); (b) a TF binds to cistrome X, and transfers to Y or Z with equal probability; (c) a TF binds to cistrome X, and transfers to Z, say; a TF binds to Y and transfers to the only free cistrome, X; (d) a TF binds to X, and transfers to Y, say; a TF binds to Y and transfers to Z, say; a TF binds to Z and transfers to X.

Our implementation model is illustrated in Fig 13, and is an implementation of this description of TF transfer between cistromes.

In our model, each gene segment has two TF binding sites: one for TF derived from the same cistrome and a secondary one for incoming TF from another cistrome. If either of these two sites is occupied at the end of a simulation step, then that gene segment will be activated and a TF product will be released to propagate the appropriate TF branching process. Fig 11 outlines four special cases of inter-cistrome communication in our model:

1. suppose that we have three interacting cistromes which share a particular gene segment (represented in the figure as an oblique red square).
2. now suppose that a TF (represented as a filled red square in Fig 11) binds to a binding site on the gene segment on Cistrome X. In our model of communication, inter-cistrome communication consists of the transfer of TFs between cistromes. Since neither Cistrome Y nor Cistrome Z has TF bound to a site in this segment yet, the TF bound at this segment in Cistrome X can transfer to the secondary binding site of the corresponding segment in Cistrome Y or Cistrome Z with equal probability.
3. suppose now, that TFs bind to appropriate sites in the gene segments in two of the Cistromes, say Cistrome X and Cistrome Y, for example. Now, Cistrome X can transfer its bound TF to either the secondary site on Cistrome Y or the binding site in Cistrome Z, with equal probability. Subsequently, any TF bound to the site in Cistrome Y can transfer to the secondary binding site in Cistrome X or that in Cistrome Z (should it not already be occupied),
4. finally, suppose that a TF binds the gene segment in all three cistromes. In this instance, the TF bound in Cistrome X can also transfer to the equivalent gene segment in Cistrome Y or Cistrome Z should their secondary TFBS not be already occupied. Cistrome Y can then transfer its bound TF to the secondary site on Cistrome X or Cistrome Z (if it is not already occupied). Finally, Cistrome Z can transfer its bound TF to the secondary site on either Cistrome X or Cistrome Y (depending on which is currently unoccupied).

Alternatively, we could have assumed that if a segment in Cistrome X has a TF molecule resulting from branching within Cistrome X bound to it, then no TFs resulting from branching in other cistromes can be accepted by the segment in Cistrome X. However, we have decided to reject this description of the system as in the biological system, communication between cistromes will occur via shared gene segments and these segments will therefore have binding sites for both the TFs concerned.

This model has limitations from the point of view of studying biologically relevant systems, because we have deferred the inclusion of destructive interference between branching processes to a later increment in order to permit the acquisition of a fuller understanding of this simplified representation of the system prior to adding another layer of complexity to the model. We also, at this point, acknowledge that another equally valid assumption about inter-cistrome communication would be that a TF transfer cannot occur between cistromes if the destination cistrome already has any TF bound to a site in the segment under consideration. i.e. in Fig 11C, Cistrome X would not be able to transfer its bound TF to Cistrome Y’s second TFBS. We believe that this alternative model will not substantially alter the qualitative nature of the results obtained from the simulation and we mean to undertake this verification prior to any further expansion of our computational model.

c-Myc is a TF that is connected with approximately 30,000 target areas throughout the genome. Thus, c-Myc represents an evolutionarily ancient undercurrent that underpins circuitry self-organization and cell behaviour in many different ways [41, 42]. c-Myc overlaps core pluripotent TF cistromes to roughly the same extent as they overlap each other (refer to Tables 1 and 2), but with m_crit_ (4) being half that for the Nanog cistrome (8) and lower than that for both Oct4 and Sox2 (6 and 7 respectively).

We tested the extended simulation by first using it to replicate the results obtained from the single cistrome simulation presented in Greaves et al. [30]. We then ran simulations in which two or more cistromes were coupled under a variety of starting conditions, e.g. one or more saturated cistromes coupled to an initially dissipated cistrome.

Visualisation of results was carried out using R scripts to present plots of the proportion of ‘red’ segments activated at a given timestep or the proportion of ‘red’ segments activated at the end of the simulation (t = 1,000) against the value of the branching parameter, *m*.
